# Improvement of phonemic fluency following leftward prism adaptation

**DOI:** 10.1038/s41598-021-86625-0

**Published:** 2021-03-31

**Authors:** Patrizia Turriziani, Gabriele Chiaramonte, Giuseppa Renata Mangano, Rosario Emanuele Bonaventura, Daniela Smirni, Massimiliano Oliveri

**Affiliations:** 1 Department of Psychology, Educational Sciences and Human Movement, University of Palermo, Viale delle Scienze, Ed. 15, 90128 Palermo, Italy; 2 NeuroTeam Life and Science, Palermo, Italy

**Keywords:** Attention, Cognitive control

## Abstract

Anatomo functional studies of prism adaptation (PA) have been shown to modulate a brain frontal-parieto-temporal network, increasing activation of this network in the hemisphere ipsilateral to the side of prism deviation. This effect raises the hypothesis that left prism adaptation, modulating frontal areas of the left hemisphere, could modify subjects’ performance on linguistic tasks that map on those areas. To test this hypothesis, 51 healthy subjects participated in experiments in which leftward or rightward prism adaptation were applied before the execution of a phonemic fluency task, i.e., a task with strict left hemispheric lateralization onto frontal areas. Results showed that leftward PA significantly increased the number of words produced whereas rightward PA did not significantly modulate phonemic fluency. The present findings document modulation of a language ability following prism adaptation. The results could have a huge clinical impact in neurological populations, opening new strategies of intervention for language and executive dysfunctions.

## Introduction

Prism adaptation (PA) is a form of visuomotor adaptation to displaced vision and it has been shown to modulate a wide range of behaviors in addition to the well-known application in patients with right hemispheric lesion and spatial neglect (for review^1–3^).

The majority of observations indicate that prism adaptation acts both on space representation as well as on other features interacting with it. For example, in healthy subjects leftward PA induces a sort of left minineglect, counteracting the physiological leftward bias called pseudoneglect^4,5^. PA cognitive effects have also been reported in visual search^6^, endogenous and/or exogenous orienting of attention^7^, spatial/temporal representation^8–14^, visually guided actions^15^, auditory representation^16^, chronic pain^17^, constructional disorders^18^ and reward-based learning^19^. Lateralized effects of PA have been reported to be dependent on the age, being more evident in young adults^20^.

Visuomotor adaptation elicited by PA can also induce modulation of frontal areas ipsilateral to prism deviation. Magnani et al.^11^, in a study using paired-transcranial magnetic stimulation (TMS) in healthy subjects, first reported modulation of excitatory brain circuits on the motor cortex specific to the direction of the visual shift induced by prismatic lenses: left-deviation PA increased excitation of the left motor cortex, while right- deviation PA increased excitation of the right motor cortex, as tested with the amplitude of motor evoked potentials.

Bracco et al.^21^ reproduced these findings in a study combining TMS, transcranial direct current stimulation (tDCS) and PA in healthy subjects. Prism adaptation increased excitability of the motor cortex ipsilateral to the deviation, as tested with TMS, in a manner similar as anodal tDCS did. The combination of the two interventions (i.e., PA and anodal tDCS) induced homeostatic plasticity effects, reducing motor cortical excitability. The same research group^22^ showed that prism adaptation induces an increase of the power of beta oscillations in the frontal areas of the hemisphere ipsilateral to the optical deviation during motor preparation but not visual attention tasks.

These findings may suggest that prism adaptation can strengthen the activation of a brain network ipsilateral to the deviation, with effects that could have an impact on the cognitive functions subserved by that network. This view suggest that left PA could modulate subjects’ performance on linguistic tasks recruiting left hemispheric areas. Related to this, left PA has been reported to activate the left dorsal attentional system and to increase interhemispheric inhibition from the left to the right hemisphere^23^. A role of increased right hemispheric inhibition in phonemic fluency tasks has been reported with rTMS in healthy subjects^24^. Moreover, PA reduces connectivity between the Default Mode Network and the inferior frontal gyrus^25^, thus increasing activation of the inferior frontal gyrus during specific tasks. The inferior frontal gyrus is involved in different components of phonological fluency, including phonological working memory and the motor articulatory processes associated with it, through the connections of area 44 with premotor cortex^26^. These inter- and intra-hemispheric connectivity changes could be associated to boosting of phonemic fluency following left PA.

In the present study, we tested these predictions by investigating the effects of left vs. right PA in modulating phonemic fluency tasks. We chose to investigate phonemic fluency because it shows a strong left hemispheric lateralization in frontal areas^27^ and it has been studied with other, neuromodulatory, techniques^24^.

Phonemic fluency tasks require search, access, selection, retrieval and pronunciation of as many words as possible in a restricted time, based on a predefined criterion of a target letter. Therefore, fluency tasks are included in many neuropsychological batteries in that they probe cognitive functions at the interface between language and executive processing. As such, phonological fluency can be impaired in a variety of clinical populations, including aphasia and dementia^28–30^.

We predicted that adaptation to a leftward optical deviation should increase subjects' performance compared to adaptation to rightward optical deviation and to no adaptation conditions.

## Results

Both leftward prism adaptation (L-PA) and rightward prism adaptation (R-PA) groups underwent a neuropsychological assessment to exclude that the results on phonemic fluency task post-PA were correlated to other cognitive functions.

There was no significant difference in the performance of the L-PA and R-PA groups on the cognitive baseline tasks: Digit Span (forward (F_1,28_ = 0.24, p = 0.78; backward F_1,28_ = 1.35, p = 0.26), SDMT (F_1,28_ = 0.22, p = 0.64), MFPT (F_1,28_ = 0.85, p = 0.36), Stroop test (F_1,28_ = 1.70, p = 0.21), RAPM (F_1,28_ = 0.68, p = 0.32).

### Effects of prism adaptation on phonemic fluency task

To compare the verbal fluency of participants at baseline (pre-PA), a one-way ANOVA with Condition (L-PA, R-PA, no-PA) variable was performed on the number of words produced in the phonemic fluency tasks. The analysis did not show significant differences (F_2,48_ = 0.04, p = 0.95, ηp^2^ = 0.002) suggesting that performance on the phonemic fluency task was similar for the three groups (L-PA = 46.73; R-PA = 46.09; no-PA = 46.57).

To investigate whether leftward and rightward PA differently modulate the phonemic fluency, a 3 × 2 repeated measures ANOVA was conducted on the number of words produced, with the variables Session (pre-PA, post-PA) as within subject factor and Condition (L-PA, R-PA, no-PA) as between subjects factor.

**Figure 1:**
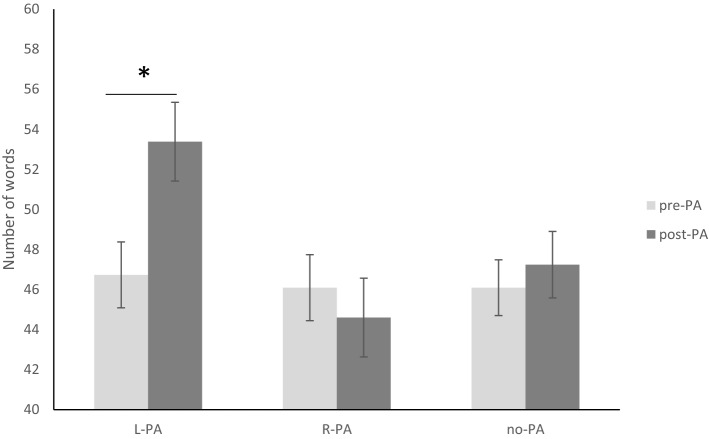
Phonemic fluency performance before and after prism adaptation (pre-PA, post-PA) across groups (L-PA group, R-PA group and no PA group). L-PA significantly improves the performance on phonemic fluency task.

The ANOVA revealed a significant effect of Session (F_1,48_ = 5.28, p = 0.02, ηp^2^ = 0.09) indicating an overall increase in the words produced in the post PA session. The main effect of Condition (F_2,48_ = 2.15, p = 0.12, ηp^2^ = 0.078) did not reach statistical significance. As shown in Fig. 1, there was a significant Session × Condition interaction (F_2,48_ = 7.64; p = 0.001, ηp^2^ = 0.24). L-PA significantly increased the number of words produced as compared with baseline (pre-PA = 46.73 vs. post-PA = 53.38; p = 0.003, ηp^2^ = 0.48). In contrast, R-PA did not significantly modulate phonemic fluency as compared with baseline (pre-PA = 46.09 vs. post-PA = 44.6, p = 0.34, ηp^2^ = 0.06). The number of words produced following left PA was higher than that following right PA (p < 0.01).

Additionally, we investigated whether PA affected the grammatical class of the words (nouns or verbs) produced in the phonemic fluency task.

An ANOVA was performed on the number of nouns and verbs produced in the phonemic fluency task, with Session (pre-PA, post-PA) and Type of word (noun, verb) as within-subjects’ factors and Condition (L-PA, R-PA, No-PA) as between-subjects factor. The analysis revealed a significant main effect of Type of word (F_1,28_ = 468.61, p = 0.000, ηp^2^ = 0.94) indicating the production of higher number of nouns (mean = 43.33) than verbs (mean = 5.58). The Session × Type of word (F_1,28_ = 10.87, p = 0.003, ηp^2^ = 0.28), Session × Condition (F_1,28_ = 4.84, p = 0.03, ηp^2^ = 0.14) and Session × Type of word × Condition (F_1,28_ = 5.73, p = 0.02, ηp^2^ = 0.17) interactions were significant.

L-PA increased the number of produced nouns (pre-PA = 42.33 vs. post-PA = 50.33, p = 0.003, ηp^2^ = 0.48) but not of verbs (pre-PA = 5.80 vs. post-PA = 6.0, p > 0.05, ηp^2^ = 0.06). R-PA did not modulate nouns (pre-PA = 40.13 vs. post-PA = 40.33, p = 0.92, ηp^2^ = 0.08) and verbs (pre-PA = 5.80 vs. post-PA = 5.50, p = 0.95, ηp^2^ = 0.01) fluency.

The Session (F_1,28_ = 3.19, p = 0.08, ηp^2^ = 0.10) and Condition (F_1,28_ = 2.29, p = 1.14, ηp^2^ = 0.76) main effects and Type of word × Condition (F_1,28_ = 2.54, p = 1.22, ηp^2^ = 0.08) interaction did not reach statistical significance.

These findings indicate that leftward PA increases the phonemic fluency of nouns but not of verbs whereas rightward PA does not modify the production of any type of words.

To investigate whether leftward or rightward PA differently modulate the motor articulatory component involved in linguistic tasks, an ANOVA with the variables Session (pre-PA, post-PA) as within subject factor and Condition (L-PA, R-PA, no-PA) as between subjects factor was conducted on the mean number of syllables (number of syllables/number of words) produced in the phonemic fluency task.

The ANOVA did not reveal main effects of Session (F_1,28_ = 1.88, p = 0.18, ηp^2^ = 0.06) and Condition (F_1,28_ = 0.18, p = 0.67, ηp^2^ = 0.006). A significant Session × Condition interaction (F_2,48_ = 4.71, p = 0.038, ηp^2^ = 0.14) was found, revealing that L-PA significantly increased the number of syllables produced (pre-PA = 2.77 vs. post-PA = 2.84, p = 0.02, ηp^2^ = 0.52). R-PA did not significantly modulate the number of syllables (pre-PA = 2.76 vs. post-PA = 2.73, p = 0.34, ηp^2^ = 0.01).

These findings indicate that leftward PA increases not only the absolute number of words produced but also the production of words formed by a greater number of syllables.

### Prism adaptation

#### Error reduction

The ANOVA showed a significant effect of Condition (F_1,28_ = 47.85, p = 0.0001, ηp^2^ = 0.20) and a significant Session × Condition interaction (F_2,48_ = 38.07, p = 0.0001, ηp^2^ = 0.62). The Session main effect was not significant (F_1,29_ = 1.03, p = 0.34, ηp^2^ = 0.03). Post-hoc analyses showed that, for both groups, the pointing displacement in the pre-exposure condition was significantly different from that in the early-exposure-condition (L-PA p = 0.001, R-PA p = 0.006).

Conversely, due to subjects’ adaptation to prismatic deviation, no differences were found between visible pointing in the pre-exposure and late exposure conditions, neither in the L-PA (p = 0.99) nor the in the R-PA (p = 0.99) group.

#### Aftereffect

The ANOVA revealed significant effects of Condition (F_1,29_ = 219.57, p = 0.000, ηp^2^ = 0.88) and Session × Condition interaction (F_1,48_ = 192.54, p = 0.000, ηp^2^ = 0.86). Session main effect was not significant (F_1,29_ = 3.28, p = 0.08, ηp^2^ = 0.10).

**Figure 2:**
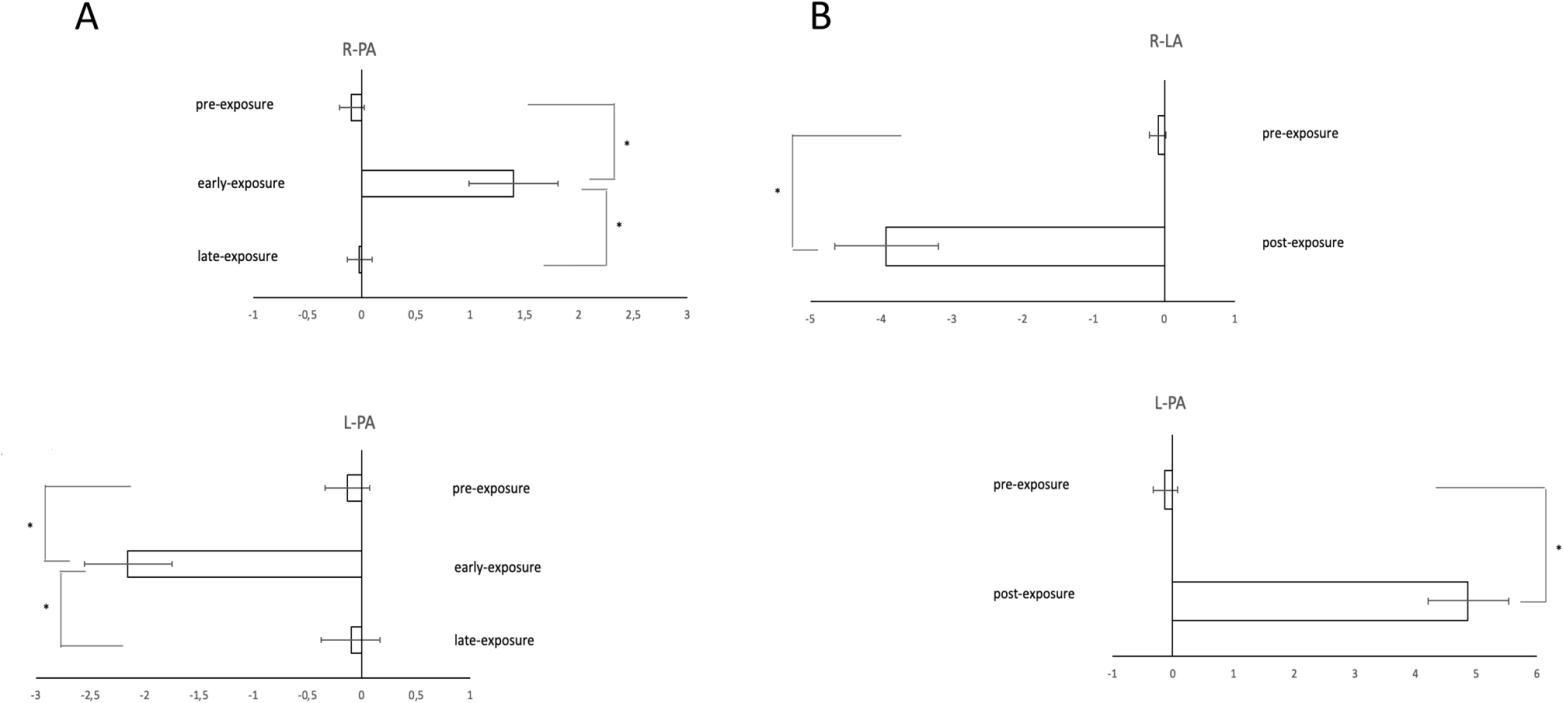
Prism adaptation parameters: (**A**) error reduction for R-PA (top) and L-PA (bottom); (**B**) aftereffect following R-PA (top) and L-PA (bottom). The values indicate mean pointing displacement in the four experimental conditions across groups. L-PA = leftward prism adaptation group; R-PA = rightward prism adaptation group; Error bars = standard error of mean; *p < 0.05. Negative values indicate leftward pointing displacement, positive values indicate rightward pointing displacement.

The presence of aftereffect was confirmed by a significant difference between pre-exposure and post-exposure in both the L-PA (p = 0.0001) and the R-PA (p = 0.0001) groups (Fig. 2).

## Discussion

The present study was aimed at investigating the effects of leftward vs. rightward PA in a phonemic fluency task.

The main results show that leftward PA is associated with improved phonemic fluency performance in healthy subjects when compared with either baseline (i.e., no optical deviation) or rightward PA. Improved phonemic fluency was evident either in terms of the number of words produced and in the number of syllables for each word. The increase in phonemic fluency following leftward PA was mainly evident for the grammatical category of nouns.

These results are unlikely to be explained by practice effects. Parallel forms of the task were used in baseline and post-PA sessions, although one target letter was common in the two fluency tasks; moreover, the control condition in the no-PA group failed to document significant increases in phonemic fluency performance across repeated sessions.

To our knowledge, this is the first study documenting facilitation of a linguistic task by prism adaptation, i.e., a procedure traditionally associated with modulation of spatial cognition or cognitive functions linked to spatial components.

According to some findings, suggesting that prism adaptation possibly increases excitability of frontal and parietal areas ipsilateral to the deviation side^11,21,22^, we may interpret the present results as probably reflecting activation of left hemispheric brain regions that are also associated with phonemic fluency tasks. In this field, neuroimaging and neuropsychological studies show that phonemic fluency recruits a left lateralized network including inferior frontal gyrus, motor cortices, anterior cingulate, temporal regions, superior parietal cortex, hippocampus, thalamus and cerebellum^31–36^. All these areas are part of a dorsal language network^37^ encompassing the left fronto-temporal arcuate fasciculus^38^, a finding consistent with the articulatory component of the phonemic fluency tasks. On the other hand, the motor articulatory component in linguistic tasks is associated with recruitment of motor cortical circuits^39^. The increase in the number of syllables produced for each word is also consistent with the recruitment of frontal motor areas^40^.

The literature shows modulation of other brain regions, in addition to frontal ones, by prism adaptation. Neuroimaging and neurophysiological studies support the idea that PA affects the visual attention and sensorimotor networks, including the parietal cortex and the cerebellum^41–44^. The activation of the parietal cortex and the cerebellum has been related to error reduction and realignment during prism adaptation. The anterior cingulate cortex is also activated in an early error-correcting phase^43^. Interestingly, parietal cortex and cerebellum are also activated during phonological fluency tasks^45,46^.

It is therefore possible that phonological fluency modulation is also controlled by the parieto-cerebellar network, activated during the spatial realignment.

The grammatical class effect encountered in phonemic boosting following leftward PA, with greater production of nouns than verbs, could depend on different factors. A neuroanatomical account posits that verb processing is mainly supported by the left frontal cortex while noun processing is supported by left temporal regions^47–50^. On the other hand, other evidence suggests that left frontal, parietal and temporal areas are similarly correlated with the noun and verb processing^51–53^.

Another possibility is linked to differential effects of PA on neural oscillations. In fact, while PA increases beta power in motor cortices ipsilateral to prismatic deviation^22^, verb retrieval is associated with beta suppression in motor areas^54^.

Modulation of a phonemic fluency task by leftward prism adaptation fits the general idea that cognition is grounded on sensorimotor interactions.

A rTMS study^24^ showed that low-frequency rTMS of the right inferior frontal gyrus increased subjects’ performance in phonological fluency tasks. The results were interpreted as reflecting plastic neural changes in the left lateral frontal cortex induced by low frequency rTMS, suppressing interhemispheric inhibitory transcallosal interactions. Interestingly, an electrophysiological study reported that leftward PA increases transcallosal interhemispheric inhibition from the left to the right primary motor cortex^55^. The results of the present study may, therefore, be also associated to modulation of transcallosal inhibition, with a reduction of activity of homologous regions of the right hemisphere, as in the reported rTMS study^24^.

Previous findings reported that rightward prism adaptation does not produce significant cognitive changes in healthy subjects^7,56–63^, (but see^22,64^ for neurophysiological changes of brain activities in healthy adults). The authors interpreted this asymmetry of prism adaptation effects as related to the right hemisphere dominance in visual attention networks^65,66^. This dominance would explain the phenomenon of leftward attentional bias called pseudoneglect. Indeed, leftward PA can counteract pseudoneglect, while rightward PA would be less efficient in shifting attention further towards the left hemispace. Therefore, one may think that the selective effects of leftward optical deviation on phonemic fluency could also be linked to the modulation of spatial factors selectively in this condition. On the other hand, since sensorimotor aftereffect and cognitive effect act at different levels, they are not consistently reported together, as suggested by other experiments exploring the effects of leftward PA in both healthy subjects and neurological patients. Schintu et al.^19^ reported modulation of left hemisphere dopaminergic activity in healthy subjects following leftward PA, without differences in the amount of sensorimotor effects induced by leftward vs. rightward PA. Frassinetti et al.^13^ first documented underestimation of time perception following leftward PA, a finding consistently replicated in other studies and correlated with the amount of sensorimotor effects^8,10,11,14,20^. In neurological patients, leftward PA has been shown to reshape visuospatial representation in left brain damaged patients with right neglect^67–69^. In other cases, spatial after-effects following leftward PA were reduced^70^ in patients with left brain damage.

The influence of spatial components on linguistic representations has been reported in the literature. Turriziani et al.^71^ described attentional representational biases in semantic judgments in healthy subjects, similar to those observed for the processing of space and numbers. Spatial manipulation of semantics was linked to the activation of specialized attentional resources located in the left hemisphere, and it was selectively modulated by left parietal rTMS. One could argue that there could be an influence of spatial factors also in the phonemic fluency task. This task requires to produce as many words as possible in a restricted time based on the predefined criterion. A leftward spatial bias has been reported for mental representations of alphabet lines. This bias is counteracted by leftward but not rightward PA^72^. Therefore, assuming that the representation of alphabet letters could be spatially organized in a left-to-right pattern, it could be hypothesized that in the present study leftward PA has shifted attention to the right space and facilitated focusing of attention to the ending letter targets (i.e., “S”). Although the hypothesis is intriguing for future, at present it only remains speculative and further, dedicated, studies, will be necessary to test this prediction.

If confirmed and extended to clinical populations of neurological patients, the present findings could help to devise a novel type of rehabilitation approach for cortical dysfunctions involving the left hemisphere. In this field, since fluency tasks lie at the interface between language and executive functions, and can be impaired in numerous neurological disorders, this behavioral rehabilitative approach could have a huge clinical impact for a variety of disorders. On the other hand, future application in patients should take into account the different interactions of side of PA with side of cerebral lesion and age of the patients^8,9,12^.

## Methods

### Subjects

Fifty-one healthy females (mean age: 24.8 ± 2.4 years) volunteered to participate in this experiments. All participants were Psychology students, native Italian speakers, right-handed, had a normal or corrected-to-normal vision and reported no history of neurological or psychiatric disease.

Thirty subjects were randomly allocated in the experimental group (mean age: 25.04 ± 2.5 years). Participants were assigned to a leftward Prism Adaptation group (L-PA; n = 15; mean age = 23.69 ± 1.88 years) or a rightward Prism Adaptation group (R-PA; n = 15; mean age = 23.86 ± 2.59 years). Participants handedness was assessed using the Edinburgh Handedness Inventory^73^.

In the control group, there were 21 right-handed healthy participants (mean age = 25.04 ± 2.51 years).

All subjects gave written informed consent for participation in the study that was approved by the ethical committee of the University of Palermo (approval n. 25/2020). The experiments were done in accord to the principles of Declaration of Helsinki.

### Neuropsychological assessment

The experimental group underwent a neuropsychological evaluation. Digit Span forward and backward^74^, Symbol Digit Modalities Test^75^ (SDMT), Modified Five Point Test^76^ (MFPT); a short version of the Stroop Colour-Word Test^77^, Raven’s Advanced Progressive Matrices^78^.

### Phonemic fluency tasks

Two phonemic fluency tasks, standardized for the Italian population, were used^79,80^. Both tasks require participants to generate as many words as possible starting with a given letter within 1 min, excluding proper nouns and words differing only for the suffix. Thus, the dependent variable was the number of words generated in 1′. In one of the two phonemic fluency tasks, the 3 letters used were “F” “A” “S”. In the second task, the 3 letters used were “F” “P” “L”.

### Prism adaptation procedure

The procedure for PA was similar to that adopted in previous studies^9,12,13,20^.

For PA, prisms of 20 prismatic diopters, inducing a leftward or rightward shift of the visual field depending on the rotation of the lenses, were used.

During PA, subjects were seated at a table in front of a box (height = 30 cm, depth = 34 cm at the center and 18 cm at the periphery, width = 72 cm) that was open on the side facing the subjects and on the opposite side, facing the experimenter. The experimenter placed a visual target (a pen) at the distal edge of the top surface of the box, in one of three possible positions (randomly determined on each trial): a central position (0°), 21° to the left of the center, and 21° to the right of the center. Subjects were asked to keep their right hand at the level of the sternum and to point toward the pen using the index finger of the same hand; the experimenter recorded the end position of the subject's pointing direction.

In the invisible pointing trials, the arm was totally covered by a black sheet and the subjects did not see any part of the trajectory of the arm.

In the visible pointing trials, the arm was covered only in the proximal part and the subjects could see the last third of the trajectory of the pointing movement.

The pointing task was performed in three experimental conditions: pre-exposure, exposure and post-exposure. In the pre-exposure condition, 60 trials were administered, 30 in visible pointing and 30 in invisible pointing. In the exposure condition, 90 trials in visible pointing were administered; subjects wore prismatic lenses that induced a 20° shift of the visual field to the right or to the left. In the post-exposure condition, 30 trials in invisible pointing were administered immediately after removal of the prisms^20^.

All the trials were equally and randomly distributed in the three marked positions of the panel.

### Experimental procedure

**Figure 3:**
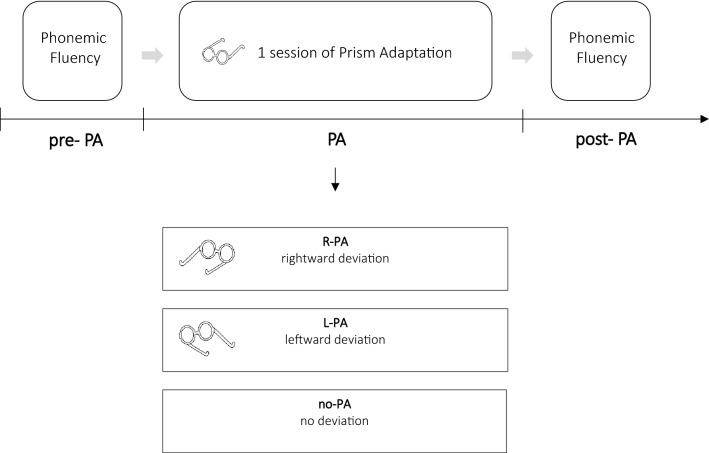
Schematic representation of the experimental design.

Both the L-PA and the R-PA groups and the control group participated in two testing sessions over two separate days, with an interval of 7 days between sessions (Fig. 3).

In the first testing session, the two experimental groups were given the cognitive baseline tasks and the phonemic fluency task (FAS or FPL).

In the second testing session, the two experimental groups were first administered the PA procedure (L-PA or R-PA), immediately followed by one of the two phonemic fluency tasks.

The control group was administered one of the two phonemic fluency tasks (FAS or FPL) in the first testing session. In the second testing session, the control group was administered the other fluency task.

The order of administration of the two phonemic fluency tasks was counterbalanced across groups and randomly assigned.

### Statistical analysis

#### Phonemic fluency task

Behavioral data were analyzed with an ANOVA, with Condition (L-PA, R-PA, No-PA) as between-subjects factor and Session (pre-PA, post-PA) as a within-subjects factor.

Post-hoc analyses were conducted with Tukey's test. Effect size is reported as partial eta square.

#### Prism adaptation

##### Error reduction

To verify whether subjects adapted to prism deviation, showing an error reduction following rightward or leftward deviation, we compared their displacement measure in the pre-exposure (visible pointing) condition with that of the first three (early- exposure condition) and the last three trials (late-exposure condition) of the exposure condition (more details on this procedure can be found in^20^). A difference between a pre-exposure condition and the early-exposure condition is expected due to the rightward or leftward displacement induced by prism exposure. On the other hand, no difference is expected between the pre-exposure and the late-exposure condition in the assumption of an almost perfect error reduction. The dependent measure in this analysis was the mean displacement (expressed as degrees of visual angle) of subjects' visible pointing. An ANOVA was conducted with Group (L-PA; R-PA) as between-subjects and Condition (pre-exposure, early-exposure and late-exposure) as within-subjects variable.

Whenever necessary, post hoc comparisons were conducted using Tukey's test. Effect size is reported as partial eta square.

##### Aftereffect

We compared the subjects' displacement in the invisible pointing in the pre-exposure and post-exposure conditions. If, after prism exposure, subjects point to the direction opposite the displacement induced by the prism, a difference is expected between the pre- and the post-exposure conditions (aftereffect). The dependent measure was the mean displacement (expressed in degrees of visual angle) of the subjects' invisible pointing responses in the pre-exposure condition and the post-exposure condition^20^. An ANOVA was conducted with Condition (L-PA; R-PA) as between-subjects and Session (pre-exposure, post-exposure) as within-subjects variable. Whenever necessary, post hoc comparisons were conducted using Tukey's test. Effect size is reported as partial eta square.
